# Probable Transmission of SARS-CoV-2 Omicron Variant in Quarantine Hotel, Hong Kong, China, November 2021

**DOI:** 10.3201/eid2802.212422

**Published:** 2022-02

**Authors:** Haogao Gu, Pavithra Krishnan, Daisy Y.M. Ng, Lydia D.J Chang, Gigi Y.Z. Liu, Samuel S.M. Cheng, Mani M.Y. Hui, Mathew C.Y. Fan, Jacob H.L. Wan, Leo H.K. Lau, Benjamin J. Cowling, Malik Peiris, Leo L.M. Poon

**Affiliations:** The University of Hong Kong, Hong Kong, China

**Keywords:** COVID-19, coronavirus disease, severe acute respiratory syndrome coronavirus 2, SARS-CoV-2, coronaviruses¸ viruses, respiratory infections, transmission, Omicron variant B.1.1.529, quarantine hotel, variant of concern, zoonoses, Hong Kong, China

## Abstract

We report detection of severe acute respiratory syndrome coronavirus 2 Omicron variant (B.1.1.529) in an asymptomatic, fully vaccinated traveler in a quarantine hotel in Hong Kong, China. The Omicron variant was also detected in a fully vaccinated traveler staying in a room across the corridor from the index patient, suggesting transmission despite strict quarantine precautions.

A new variant of severe acute respiratory syndrome coronavirus 2 (SARS-CoV-2), B.1.1.529, was identified in Botswana and South Africa in early November 2021 and was designated as variant of concern (VOC) Omicron by the World Health Organization on November 26, 2021 (*1*). As of December 1, 2021, ≈220 sequences were available on GISAID (https://www.gisaid.org), and this variant has been detected in countries in Africa and beyond since mid-November (*2*,*3*). This variant contains >30 spike protein amino acid mutations that might be associated with increased transmissibility, severity, and capacity for immune escape. With supporting evidence of epidemiologic and molecular epidemiologic findings, we report the probable transmission of Omicron in a quarantine hotel in Hong Kong, China. We also compare its mutational profile with other VOCs and variants of interest.

Two cases of infection with VOC Omicron (cases A and B) were detected in Hong Kong. Case-patient A arrived in Hong Kong from South Africa on November 11, 2021, and case-patient B arrived in Hong Kong from Canada on November 10, 2021. Both case-patients had previously received 2 vaccine doses (Pfizer-BioNTech, https://www.pfizer.com); the second dose was given on June 4, 2021, for case-patient A and on May 25, 2021, for case-patient B. Both case-patients tested negative by reverse transcription PCR (RT-PCR) for SARS-CoV-2 within 72 hours before arrival. On arrival at the Hong Kong airport, both case-patients stayed in the same quarantine hotel and had rooms across the corridor from each other on the same floor. 

Case-patient A showed a positive result for SARS-CoV-2 without symptoms on November 13, 2021 (cycle threshold [C_t_] value 18). He was hospitalized and isolated the next day. Case-patient B had mild symptoms develop on November 17, 2021. He showed a positive result for SARS-CoV-2 (C_t_ value 19) on November 18, 2021, and was hospitalized on the same day. The 2 C_t_ values indicate high viral loads. None of the 12 persons staying in nearby rooms on the same floor during the study or related hotel staff have tested positive in repeated tests for SARS-CoV-2 (*4*).

Viral genomes deduced from these 2 SARS-CoV-2‒positive cases differed only by 1 nt. Retrospective investigation, including closed-circuit television camera footage, confirmed that neither case-patient left their room during the quarantine period. No items were shared between rooms, and other persons did not enter either room. The only time the 2 quarantined persons opened their respective doors was to collect of food that was placed immediately outside each room door. The only other time they might have opened their doors would be for RT-PCRs, which were conducted in 3-day intervals. However, because these 2 case-patients arrived 1 day apart, it is unlikely that they would be tested on the same day. Airborne transmission across the corridor is the most probable mode of transmission.

We sequenced complete SARS-CoV-2 genomes from case-patients A and B (Appendix) and confirmed that these genomes were VOC Omicron (Pango lineage B.1.1.529) (Figure, panel A). Viral sequences from these 2 case-patients differed by only 1 nt. Viral sequence from case-patient A was highly similar to those of the first few reported Omicron cases identified in South Africa and Botswana (Appendix Table 1). Because many countries have just reported detection of this VOC (https://www.gisaid.org/hcov19-variants), the actual genetic diversity of this virus lineage requires further investigations.

**Figure:**
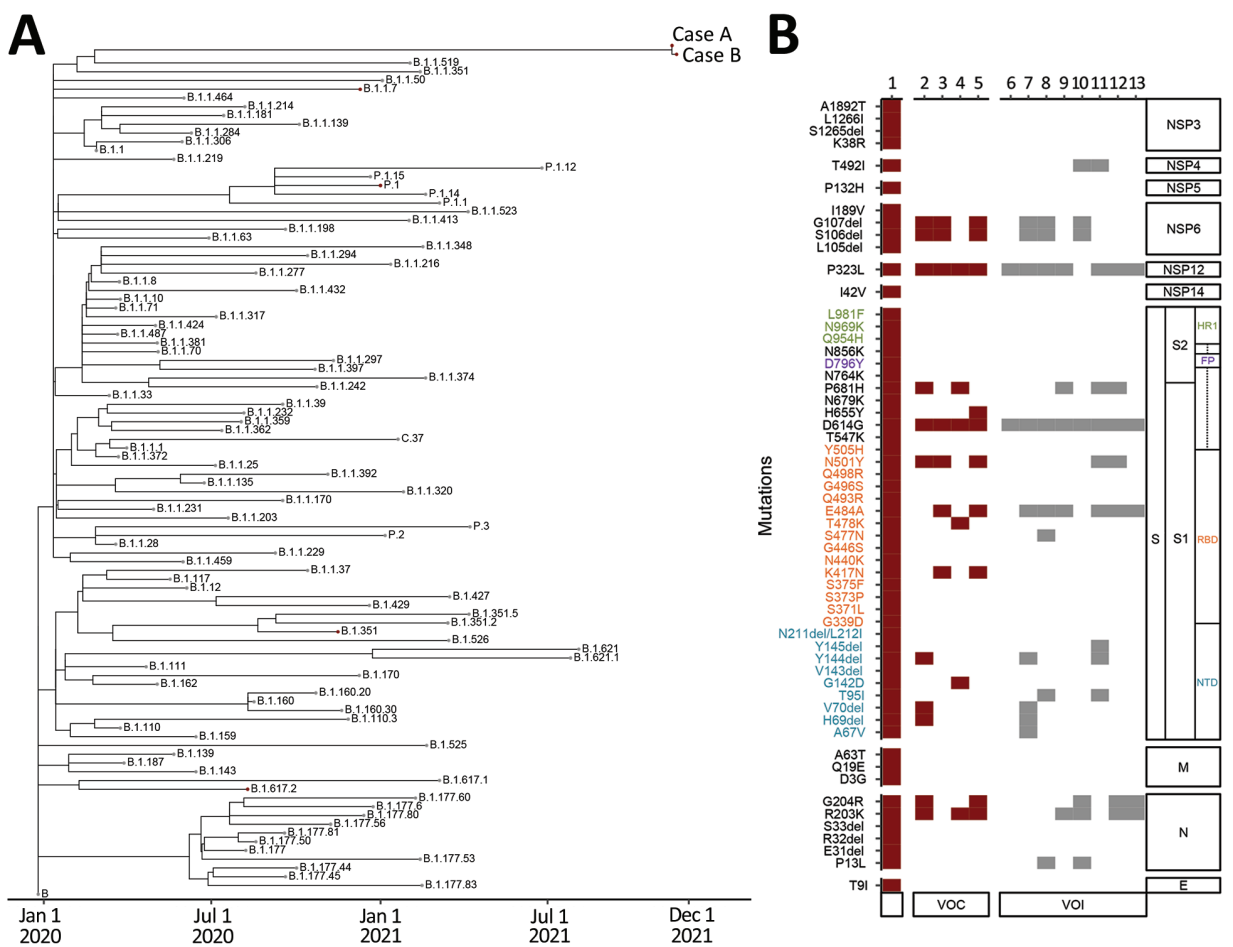
Detection of severe acute respiratory syndrome coronavirus 2 Omicron variant in 2 patients (cases A and B) in Hong Kong, China, November 2021. A) Phylogenetic time tree of Omicron nucleotide sequences using an early severe acute respiratory syndrome coronavirus sequence as a reference sequence (Wuhan-Hu-1/2019; GenBank accession no. MN908947.3). B) Comparison of Omicron variant mutations in case A to other variants; red indicates VOC and gray VOI (Appendix). Text colors indicate mutations found in NTD (blue), RBD (orange), FP (purple), and HR1 (green). Lane 1, case A; 2, Alpha (B.1.1.7); 3, Beta (B.1351); 4, Delta (B.1.617.2); 5, Gamma (P1); 6, Epsilon (B.1.427/429); 7, Eta (B.1.525); 8, Iota (B.1.526); 9, Kappa (B.1.617.1); 10, Lambda (C.37); 11, Mu (B.1.1.621); 12, Theta (P.3); 13, Zeta (P.2). E, envelope; FP, fusion peptide; HR1, heptad repeat 1; M, matrix; NSP, nonstructural protein; NTD, N-terminal domain; RBD, receptor-binding domain; S, spike; VOC, variant of concern; VOI, variant of interest.

The long branch of Omicron clade in the phylogenetic tree is attributed to the large number of mutations (Figure, panel A). Nonsynonymous mutations were identified in the spike (S)‒encoding (n = 35) and other viral protein‒encoding (n = 22) regions (Figure, panel B). Among the nonsynonymous mutations in the S protein, 43% (n = 15) were also identified in other VOCs/variants of interest, and 31% (n = 11) were found only in VOCs (Alpha, n = 6; Beta, n = 4; Gamma, n = 5; Delta, n = 4). Some of the point mutations and deletions found in other regions are not novel and can also be found in other variants at different frequencies (Appendix Table 2). Among these non-S mutations, NSP4-T492I, NSP6-S106del, NSP6-G107del, NSP12-P323L, N-P13L, N-R203K, and N-G204R are commonly found in SARS-CoV-2 variants.

The laboratory and epidemiologic features of the Omicron variant are yet to be fully characterized and cannot be determined on the basis of sequence features alone. Nonetheless, compared with other VOCs, the number of mutations found in the spike of the Omicron variant is unprecedented. This finding results in false-negative results in some diagnostic RT-PCRs specific for the S gene (*3*). Many of the mutations found in the S protein are known to alter SARS-CoV-2 antigenicity and transmissibility (*5*). The R203K and G204R mutations in the nucleocapsid protein are also associated with enhanced virus replication (*6*).

It is not known whether these detected mutations might have affected the effectiveness of existing vaccines and virus transmissibility. However, detection of Omicron variant transmission between 2 fully vaccinated persons across the corridor of a quarantine hotel has highlighted this potential concern. Further experimental characterizations and epidemiologic investigations of this newly found VOC are urgently needed. Increased precautions or additional measures might be warranted while awaiting more data.

Appendix Additional information on probable transmission of SARS-CoV-2 omicron variant in quarantine hotel, Hong Kong, China, November 2021.
