# Supplementary material for: Probable Transmission of SARS-CoV-2 Omicron Variant in Quarantine Hotel, Hong Kong, China, November 2021
Source: Emerg Infect Dis. 2022 Feb;28(2):460–2. doi: 10.3201/eid2802.212422 (PMC8798678; doi:10.3201/eid2802.212422)
Supplement: Appendix — Additional information on probable transmission of SARS-CoV-2 omicron variant in quarantine hotel, Hong Kong, China, November 2021. [file 21-2422-Techapp-s1.pdf]

Article DOI: https://doi.org/10.3201/eid2802.212422

Probable Transmission of SARS-CoV-2 Omicron Variant in Quarantine Hotel, Hong Kong, China, November 2021

Additional Methods

Sequencing

Respiratory swab samples from cases A and B were subjected to next-generation sequencing. RNA samples were sent to a World Health Organization reference laboratory at the University of Hong Kong for full-genome analyses (Institutional Review Board no. UW 20–168). We deduced near full-length genomes from the samples by using a described Illumina (https://www.illumina.com) sequencing protocol (*1*,*2*). Briefly, virus genome was reverse transcribed with multiple gene-specific primers targeting different regions of the viral genome. The synthesized cDNA was then subjected to multiple overlapping 2-kb PCRs for full-genome amplification. PCR amplicons obtained from the same specimen were pooled and sequenced by using the iSeq sequencing platform (Illumina). Sequencing library was prepared by using Nextera XT (illumine). Generated sequencing reads were mapped to a reference virus genome by using the Burrow–Wheeler Aligner (*3*), and genome consensus was generated by using iVar with the PCR primer trimming protocol (minimum sequence depth >10 and minimum Q value of 30) (*4*). The deduced sequences are available at GISAID (Accession nos. EPI_ISL_6716902 and EPI_ISL_6716890).

Phylogenetic Analysis

The 2 sequences from Hong Kong were analyzed together with a set of representative sequences from other lineages, including all sublineages under B.1.1 (Pango lineage) and all variants of concern/variants of interest lineages. The sequences were retrieved from the presubsampled prealigned open database from Nextstrain (https://docs.nextstrain.org/projects/ncov/en/latest/reference/remote_inputs.html). The maximum-likelihood phylogenies were estimated by using IQ-TREE version 2.1.3 (*5*) and the general time reversible + empirical base frequencies + FreeRate model of with number of

categories of 2 nucleotide substitution model with Wuhan-Hu-1 (GenBank accession no. MN908947.3) as the outgroup. Dating of the tree were performed by using IQ-TREE LSD2 with specifications "–date-root 2019-12-26–date-ci 100–date-options \"-l −1\"."

Mutation Analysis

The lineages defining mutations (or lineage specific mutations) for different variants of concern/variants of interest (Figure, panel B) were curated from 3 public databases (https://covariants.org/shared-mutations, https://github.com/cov-lineages/constellations, and https://outbreak.info/). Detailed analyzing scripts used in the study can be accessed in a GitHub repository (https://github.com/Leo-Poon-Lab/Detection-of-B.1.1.529-variant-in-Hong-Kong).

References

1. Choi EM, Chu DK, Cheng PK, Tsang DN, Peiris M, Bausch DG, et al. In-flight transmission of SARS-CoV-2. Emerg Infect Dis. 2020;26:2713–6. PubMed https://doi.org/10.3201/eid2611.203254

2. Sit TH, Brackman CJ, Ip SM, Tam KW, Law PY, To EM, et al. Infection of dogs with SARS-CoV-2. Nature. 2020;586:776–8. PubMed https://doi.org/10.1038/s41586-020-2334-5

3. Vasimuddin M, Misra S, Li H, Aluru S. Efficient architecture-aware acceleration of BWA-MEM for multicore systems. In: 33rd IEEE International Parallel and Distributed Processing Symposium (IPDPS), 2019. Rio de Janeiro, May 20–24, 2019. p. 314–24.

4. Grubaugh ND, Gangavarapu K, Quick J, Matteson NL, De Jesus JG, Main BJ, et al. An amplicon-based sequencing framework for accurately measuring intrahost virus diversity using PrimalSeq and iVar. Genome Biol. 2019;20:8. PubMed https://doi.org/10.1186/s13059-018-1618-7

5. Minh BQ, Schmidt HA, Chernomor O, Schrempf D, Woodhams MD, von Haeseler A, et al. IQ-TREE 2: new models and efficient methods for phylogenetic inference in the genomic era. Mol Biol Evol. 2020;37:1530–4. PubMed https://doi.org/10.1093/molbev/msaa015

Appendix Table 1. Nucleotide divergences between viral sequences of case A with other Omicron virus sequences

Reference sequence (case A)	No. nucleotide divergences*	No. nucleotide divergences in spike gene*
hCoV-19/Botswana/R40B59_BHP_3321001248/2021|EPI_ISL_6640916|2021–11–11	1	0
hCoV-19/Botswana/R40B60_BHP_3321001247/2021|EPI_ISL_6640917|2021–11–11	1	0
Case B†	1	0
hCoV-19/South_Africa/NICD-N21607-DX64624/2021|EPI_ISL_6647962|2021–11–16	1	1
hCoV-19/Botswana/R40B58_BHP_3321001245/2021|EPI_ISL_6640919|2021–11–11	2	0
hCoV-19/South_Africa/NICD-N21600-DX03569/2021|EPI_ISL_6647956|2021–11–14	2	2
hCoV-19/South_Africa/NICD-N21602-DX040380/2021|EPI_ISL_6647957|2021–11–15	2	2
hCoV-19/South_Africa/NICD-N21605-DX64490/2021|EPI_ISL_6647960|2021–11–15	3	2
hCoV-19/South_Africa/NICD-N21603-DX64204/2021|EPI_ISL_6647958|2021–11–16	4	2
hCoV-19/South_Africa/NICD-N21604-DX64219/2021|EPI_ISL_6647959|2021–11–16	6	2
USA/ID-CDC-LC0011682/2021 (B.1.1.519)	55	27
Wuhan-Hu-1/2019	54	30

*Ambiguous or deleted nucleotide regions in these published sequences are excluded in the analysis.
†Viral sequence of case B differs from that of case A by 1 nt (nt position G6167C) and this mutation cannot be found in other reported Omicron virus variant sequences.

Appendix Table 2. Nonsynonymous mutations found in VOC Omicron*

Gene	Mutation	Frequency in GISAID, %
NSP3	K38R	0.01
NSP3	V1069I	0.02
NSP3	S1265del	0.02
NSP3	L1266I	0.02
NSP3	A1892T	0.00
NSP4	T492I	46.49
NSP5	P132H	0.01
NSP6	L105del	0.02
NSP6	S106del	25.59
NSP6	G107del	25.59
NSP6	I189V	0.03
NSP12	P323L	96.94
NSP14	I42V	0.00
Spike	A67V	0.37
Spike	H69del	21.90
Spike	V70del	21.93
Spike	T95I	20.79
Spike	G142D	32.16
Spike	V143del	0.13
Spike	Y144del	21.66
Spike	Y145del	19.25
Spike	N211del/L212I	0.02/0.01
Spike	G339D	0.01
Spike	S371L	0.00
Spike	S373P	0.01
Spike	S375F	0.00
Spike	K417N	0.86
Spike	N440K	0.17
Spike	G446S	0.01
Spike	S477N	1.36
Spike	T478K	51.35
Spike	E484A	0.02
Spike	Q493R	0.01
Spike	G496S	0.01
Spike	Q498R	0.00
Spike	N501Y	24.94
Spike	Y505H	0.00
Spike	T547K	0.01
Spike	D614G	98.81
Spike	H655Y	2.32
Spike	N679K	0.10
Spike	P681H	23.51
Spike	N764K	0.01
Spike	D796Y	0.08
Spike	N856K	0.00
Spike	Q954H	0.00

Gene	Mutation	Frequency in GISAID, %
Spike	N969K	0.00
Spike	L981F	0.00
Matrix	D3G	0.08
Matrix	Q19E	0.00
Matrix	A63T	0.01
Nucleocapsid	P13L	0.65
Nucleocapsid	E31del	0.00
Nucleocapsid	R32del	0.00
Nucleocapsid	S33del	0.00
Nucleocapsid	R203K	28.70
Nucleocapsid	G204R	27.10
Envelope	T9I	0.09

*NSP, nonstructural protein.

Appendix Table 3. GISAID sequences used in this study

Accession no.	Originating laboratory	Submitting laboratory	Authors
EPI_ISL_6640916, EPI_ISL_6640917, EPI_ISL_6640919	Botswana Harvard HIV Reference Laboratory	Botswana Harvard HIV Reference Laboratory	Sikhulile Moyo, Wonderful T. Choga, Dorcas Maruapula, Keoratile Ntshambiwa, Sefetogi Ramaologa, Thongbotho Mphoyakgosi, Boitumelo Zuze, Botshelo Radibe, Legodile Kooepile, Ontlametse T. Bareng, Pamela Smith-Lawrence, Kgomotso Moruisi, Roger Shapiro, Shahin Lockman, Joseph Makhema, Mphaphi B. Mbulawa, Mosepele, Simani Gaseitsiwe
EPI_ISL_6647956 EPI_ISL_6647957 EPI_ISL_6647958 EPI_ISL_6647959 EPI_ISL_6647960 EPI_ISL_6647962	Lancet laboratory	National Institute for Communicable Diseases of the National Health Laboratory Service	D.G. Amoako, J. Everatt, C. Scheepers, A. Glass, Viana R, Mohale T.N. Ntuli, B. Mahlangu, A. Mnguni, A. Ismail, J.N. Bhiman
